# Differential expression of Caveolin-1 in hepatocellular carcinoma: correlation with differentiation state, motility and invasion

**DOI:** 10.1186/1471-2407-9-65

**Published:** 2009-02-24

**Authors:** Murat Cokakli, Esra Erdal, Deniz Nart, Funda Yilmaz, Ozgul Sagol, Murat Kilic, Sedat Karademir, Nese Atabey

**Affiliations:** 1 Dokuz Eylul University, Faculty of Medicine, Department of Medical Biology and Genetics, Inciralti- Izmir/Turkey; 2 Ege University, Faculty of Medicine, Department of Pathology, Bornova- Izmir, Turkey; 3 Dokuz Eylul University, Faculty of Medicine, Department of Pathology, Izmir, Turkey; 4 Ege University, Faculty of Medicine, Department of Surgery, Bornova- Izmir, Turkey; 5 Dokuz Eylul University, Faculty of Medicine, Department of Surgery, Izmir, Turkey

## Abstract

**Background:**

Caveolin-1 is the main component of caveolae membrane structures and has different roles during tumorigenesis in different cancer types with varying expression profiles, indicating that the role of caveolin-1 varies according to tumor type. In this study, we investigated the role and expression of caveolin-1 in hepatocellular carcinogenesis.

**Methods:**

We analyzed the expression of Caveolin-1 in 96 hepatocellular carcinoma (HCC), 29 cirrhosis, 20 normal liver tissues and 9 HCC cell lines by immunostaining and western blotting, respectively. After caveolin-1 was stably transfected to HepG2 and Huh7 cells, the effects of Caveolin-1 on the cellular motility, matrix invasion and anchorage-independent growth were studied. Also, caveolae structure was disrupted in endogenously caveolin expressing cells, SNU 449 and SNU 475 by addition of methyl-β-cyclodextrin and analyzed cellular motility and invasion.

**Results:**

In HCC cell lines, Caveolin-1 expression is correlated to differentiation and basal motility status of these cells. The percentage of Caveolin-1 positivity was found extremely low in normal liver tissue (5%) while it was increased in cirrhosis (45%) and in HCC (66%) (p = 0.002 and p = 0.001 respectively). Cav-1 expression in poorly differentiated HCC samples has been found significantly higher than well differentiated ones (p = 0.001). The caveolin-1 expression was found significantly higher in tumor cells than its peritumoral cirrhotic tissues in HCC samples (p < 0.001). Additionally, the patients with positive staining for Caveolin-1 had significantly higher portal vein invasion than those with negative staining (p = 0.02). Caveolin-1 overexpression increased motility and invasion of HepG2 and Huh7 cells. And disruption of caveolae results in a dramatic decline in both motility and invasion abilities in SNU-449 and SNU-475 cells. Furthermore, caveolin-1 overexpression resulted in down-regulation of E-cadherin while up-regulation of Vimentin. Also, it increased secreted MMP-2 and expression levels of MMP-9 and MT1-MMP. There was no significant difference in colony formation in soft agar between stable clones and parental ones.

**Conclusion:**

In conclusion, stepwise increase in Cav-1 expression in neoplastic stage with respect to pre-neoplastic stage during hepatocellular carcinogenesis and its ability to stimulate HCC cell motility and invasiveness indicate that this protein plays a crucial role in tumor progression.

## Background

Dysregulation of pleiotropic growth factors, receptors and their downstream signaling pathway components represent a central protumorigenic principle in human hepatocarcinogenesis [1]. Especially the Insulin-like Growth Factor (IGF-1/IGF-1R), Hepatocyte Growth Factor (HGF/MET), Wnt (Wnt/FZD), Transforming Growth Factor-alpha/Epidermal Growth Factor (TGF-α/EGF/EGFR) and Transforming Growth Factor-beta (TGF-β/TGF-βR) pathways contribute to proliferation, antiapoptosis and invasive behavior of hepatocellular carcinoma cells [1-4]. It has been shown that caveolae formation and/or expression of Caveolin-1 (Cav-1) affects the biological consequences of such signaling pathways via particular mechanisms including receptor internalization [5-7]. Although there is little evidence about involvement of Cav-1 expression in hepatocarcinogenesis, the role of Cav-1 is not clarified completely. Recently, it was found that Cav-1 is essential for liver regeneration and plays a crucial role in liver proliferation in response to partial hepatectomy in mice [8]. Mechanisms of liver regeneration processes have been suggested to be similar with hepatocarcinogenesis [9,10]. Generally, cell proliferation occurs in both liver regeneration and malignant tumor growth, but regeneration process is controlled stringently whereas tumorigenesis is uncontrolled.

Cav-1 is a cell surface plasma membrane protein that is necessary and sufficient for the formation of caveolae invaginations on cell membrane [11]. Caveolae and Cav-1 participate together in several cellular processes such as vesicular transport, lipid metabolism, cholesterol homeostasis, and regulation of signal transduction including cell cycle, growth and apoptosis [12]. Additionally, Cav-1 has been implicated in the pathogenesis of oncogenic cell transformation and tumorogenesis. It was shown that differences in Cav-1 expression depend on the type of tumor cells. For instance, while Cav-1 downregulation is typical for ovarian, lung, and mammary carcinomas, it is upregulated in bladder, esophagus, thyroid and prostate carcinomas [13]. Furthermore, Cav-1 represents an acquired feature that contributes to metastatic phenotype in different types of carcinomas [14,15]. A growing body of evidence suggests Cav-1 plays an essential role during development of HCC. Yokomori *et al*. reported that Cav-1 expression elevated in cirrhotic liver, while it was almost undetectable in normal liver, and it may be associated with significant reduction in nitric oxide catalytic activity in cirrhosis [16]. On the other hand, it was shown that Cav-1 expression was inactivated in HCC cell lines by aberrant methylation and it may serve as important biomarkers of malignancy [17]. These observations do not provide clear evidence about the role of Cav-1 in hepatocellular carcinogenesis. In a few studies, it was shown that caveolae functions to compartmentalize certain proteins which bind directly to Cav-1 and regulate signaling in HCC. For instance, deleted in liver cancer-1 (DLC1) and Tensin-2 proteins bind to Cav-1 and this complex interacts with Rho GTPases in caveolae to effect cytoskeletal reorganization in HCC cell lines [18]. However, the distribution and significance of Cav-1 expression in normal and cirrhotic liver and the role in hepatocellular carcinogenesis still remain poorly understood.

In this study, we aimed to identify the expression status of Cav-1 in normal, cirrhotic, and HCC tumor samples, in order to investigate the changes in Cav-1 expression during progression from normal liver to cirrhosis and HCC. We complemented these descriptive analyses by studying its *in vitro *cellular effects, following stable expression in HCC cell lines and disruption of caveolae with chemical inhibitors.

## Methods

### Cell Culture

Human hepatocellular carcinoma cell lines Huh7, HepG2, Hep3B, SK-Hep1, SNU-398, SNU-449, SNU-475, PLC/PRF-5 and Mahlavu [19] were cultured in DMEM supplemented with 10% FBS, 100 u/ml penicillin, 0,1 mg/ml streptomycin and 2 mM L-glutamine in a CO_2 _incubator at 37°C. All cell culture reagents were purchased from Biological Industries (Israel). HCC cell lines were kindly provided by Dr. Mehmet Ozturk (Bilkent University, Ankara, Turkey).

### Immunoblotting and Gelatin-Zymography

Total cell lysates were prepared from HCC cell lines with RIPA buffer (50 mM Tris-Cl pH 7.4, 150 mM NaCl, 1 mM EDTA pH 8.0, %1 NP-40, 1X protease inhibitor cocktail, 1 mM NaF, 1 mM Na_3_VO_4_). Protein concentrations were determined using BCA assay under manufacturer's instructions (Pierce, IL, USA). 50 ug of total cell lysates were boiled with Laemli buffer (Applichem, Darmstadt, Germany) and loaded onto SDS-PAGE. Resolved proteins were transferred onto PVDF membranes (Millipore, MA, USA) and immunoblotted with appropriate antibodies. Primary antibodies were purchased: Cav-1 (sc-894), calnexin (sc-11397), E-cadherin (sc-8426), MMP-2 (sc-13595), MMP-9 (sc-21733), MT1-MMP (sc-30074) from Santa Cruz (California, USA); Vimentin (550513) from BD Pharmingen (BD Biosciences, CA, USA); HRP-conjugated anti-rabbit and anti-mouse secondary antibodies from Pierce. Proteins were detected using ECL detection system (Pierce, IL, USA).

For the gelatin-zymography assays, cells were grown in serum-free medium for 24 hrs and culture supernatant was directly used for detection of secreted MMPs. Briefly, 40 ul of medium was loaded onto 7.5% polyacrylamide gel containing 1 mg/ml gelatin. The gels were washed in 2.5% Triton X-100 and then incubated in 50 mM Tris-Cl (pH 7.0) solution containing 10 mM CaCl2 and 150 mM NaCl at 37°C for 24 hrs. Following incubation, the gels were stained with 0.25% commasive-blue for 1 hr and destained with destaining-buffer (40% methanol, 20% acetic-acid) until bands become clear. All chemicals were purchased from Applichem (Darmstadt, Germany).

### Preparation of stable cell-lines

Cav-1 cDNA was amplified from human HCC cell line Mahlavu with Pfu DNA polymerase (MBI Fermentas, Ontario, Canada), cloned into pcDNA3.1/Myc-His expression vector (Invitrogen, CA, USA) using T4 DNA ligation kit (Takara Bio, Shiga, Japan). Plasmid maxipreparation kit was from Qiagen. Plasmids were controlled with sequencing (Macrogen Inc., Seoul, Korea). Constructed pcDNA3.1/CAV1 vector was transfected into HepG2 and Huh7 cells using TransFast reagent (Promega, WI, USA). Transfected cells were treated with 1000 ug/ml Geneticin (Gibco BRL, CA, USA) for HepG2 and 500 ug/ml for Huh7. After 3-weeks of incubation, Geneticin-resistant colonies were picked-up with cloning cylinders. Stable colonies were controlled for caveolin-1 expression by western-blotting.

### *In-vitro *motility and invasion assays

The motility of parental and Cav-1 transfected HepG2 and Huh7 cells in modified Boyden chambers were measured using 8 um pore size of Biocoat Cell Environment control inserts (BD Biosciences, CA, USA). Briefly, 70% confluent cells were starved over-night with DMEM containing 1% FBS. Lower chambers were filled with 750 ul of DMEM containing 10% FBS. Starved cells were trypsinized, washed in DMEM and added to upper chambers (2 × 10^6 ^cells/ml) and incubated at 37°C for 24 h. After incubation, media were removed; cells were fixed with methanol and stained with Diff Quick (Siemens Healthcare Diagnostics, IL, USA). Cells on the upper surface of each membrane were removed with cotton swabs, whereas cells that had transversed to the bottom surface of the filters were counted using brightfield microscope. Total cell numbers were counted for each wells. The ratio of Cav-1 transfected to parental migrating cells is designated on the *y *axis as "migration (-fold increase)" +/- standard deviation.

Huh7 and HepG2 cell invasion assays were performed using Matrigel Invasion Chambers (BD Biosciences, CA, USA) and quantitated as described for cell migration across uncoated membranes. Briefly, cells were seeded at 2 × 10^6 ^cells/ml in serum-free DMEM + 0,1%BSA and placed into 24-wells culture plates containing DMEM + 5% FBS for 24 h at 37°C. The ratio of Cav-1 transfected cells to parental invading cells is designated on the *y *axis as "invasion (-fold increase)" +/- standard deviation. All experiments were repeated at least three times.

For the disruption of caveolae structure, a cholesterol depleting-agent methyl-β-cyclodextrin (MBCD) was used as described previously [20]. Briefly, near-confluent cells were washed with PBS and incubated 1 hour with serum-free DMEM containing 10 mM MBCD (Sigma-Aldrich, MO, USA). After treatment, cells were trypsinized, counted and used for motility and invasion experiments.

### Colony formation in soft-agar

The anchorage-independent growth of parental and Cav-1 expressing Huh7 and HepG2 cells were determined by assaying colony formation in soft-agar as described by Koleske *et al*. with some modifications [21]. Briefly, single cell suspension of parental and caveolin transfected cells were prepared by trypsinization and homogenization. Cells were suspended in DMEM containing 10% FBS as 5 × 10^5 ^cell/ml. Cells were plated onto each well of a 24-well plate at a density of 2500 cells/well in DMEM containing 10% FBS and 0.4% low melting point agarose (Sigma, MO, USA) on a base layer of 0.6% low melting point agarose (Sigma, MO, USA). The medium was refreshed every 3 days. The number of colonies was counted and photographed after 2–3 weeks of incubation at 37°C. The assays were performed in triplicate and repeated at least three times.

### Tissue samples and immunohistochemistry

Tissue samples were obtained from 95 patients with HCC in cirrhotic background and other 29 patients with cirrhosis who underwent complete resection without any preoperative treatment in Dokuz Eylul University and Ege University Hospitals in Izmir, Turkey. Liver biopsies taken as a part of the evaluation of potential donors for living donor liver transplantation at Ege University Hospital were used as control normal liver tissue (n = 20). The study was approved by the Ethics Committees of both Dokuz Eylul and Ege University Medical Schools. Written informed consents from patients were obtained before liver transplantation or liver biopsy sampling. All tissue samples were fixed in formalin and embedded in paraffin, standard 5 μm tissue sections were obtained and stained with hematoxylen-eosin and examined by light microscopy. The histopathological diagnosis of all patients was carried out by the WHO histopathological classification of liver and intrahepatic bile ducts [22]. Each slide was independently evaluated by two pathologists unaware of clinicopathological characteristics of the tumor.

For immunostaining, paraffin embedded tissues were deparaffinized in xylene and were rehydrated. Immunostaining was performed using an automated immunohistochemical stainer according to the manufacturer's guidelines (IVIEW™ DAB, BenchMarkXP, Ventana, USA). The antigen retrieval step was included in the automated programme. Endogenous peroxidase activity was blocked by 3% H_2_O_2 _for 4 minutes at 37°C. Endogenous avidin biotin activity was blocked by the avidin-biotin block solution (DAKO, Denmark). Sections were incubated with anti-caveolin-1 (sc-894) antibody at 1:100 dilution. The reaction product was developed using 3,3-diaminobenzidine tetrahydrochloride (DAB) and sections were counterstained with hematoxylin. Sections were then evaluated by two pathologists (FY, DN) who were blinded to the clinical information on patients. Positive staining was defined as membranous and/or cytoplasmic expression. The extent of caveolin expression was semiquantitatively graded as 0 (less than 10% of the cells), 1+ (positivity between 10%–50% of cells), and 2+ (more than 50% of cells). The intensity of caveolin expression was semiquantitatively graded as 0, 1 or 2. Additionally, profile of caveolin expression was investigated by comparing staining intensities in the tumor cells with respect to its surrounding tissue and graded as above.

### Statistical analysis

Statistical analysis of obtained data was performed using the SPSS-Software. Correlation analysis between Cav-1 expression and clinicopathological data including information on TNM classification, histopathological grading, number of tumor nodules, tumor size, presence of venous invasion and tumor etiology was performed using cross-tables applying χ^2 ^test. A *P*-value of ≤ 0.05 was considered as indicative of statistical significance for the tests used.

## Results

### Caveolin-1 expression is correlated with differentiation and motility status of HCC cell lines

**Figure 1:**
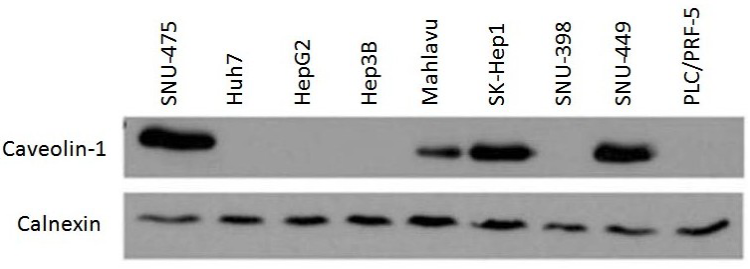
**Expression of Cav-1 protein in HCC cell lines**. SNU-475, Mahlavu, SK-Hep1 and SNU-449 express caveolin-1 while Huh7, HepG2, Hep3B, SNU-398 and PLC/PRF-5 do not express it (Calnexin was used as internal control for western blotting).

We studied Cav-1 expression in HCC cell lines by western blotting. Caveolin-1 was expressed in 4 cell lines (SNU-449, SNU-475, SK-Hep1, and Mahlavu), but not in 5 others (SNU-398, HepG2, Hep3B, PLC/PRF-5 and Huh7), as shown in Figure 1. When we analyzed differentiation status of these HCC cell lines according to HNF1α, 3α, 3β, 4α and 6 expressions, HepG2, Hep3B, PLC/PRF-5 and Huh7 formed a group of well differentiated cell lines, whereas others formed another subgroup of poorly differentiated cell lines (E. Erdal *et al*.; unpublished data). Thus, Cav-1 expression was closely related to the differentiation status of these cell lines. It was not expressed in well-differentiated cell lines, whereas 4 out of 5 poorly differentiated cell lines displayed high levels of Cav-1 expression (Table 1). Moreover, motility capacity of these HCC cell lines [23] was positively correlated with Cav-1 expressions as shown in Table 1.

**Table 1:** Differentiation status and caveolin-1 expressions of HCC cell lines.

*Cell Lines*	*Differentiation Status*	*Motility Status*	*Caveolin Expression (protein level)*
Huh7	well	-	-
Hep3B	well	-	-
HepG2	well	-	-
PLC/PRF-5	well	ND*	-
SNU-398	poorly	-	-
SNU-475	poorly	+	+
SNU-449	poorly	+	+
SK-Hep1	poorly	+	+
Mahlavu	poorly	+	+

*Not Determined

### Increased expression of Caveolin-1 in cirrhotic and HCC tissue samples compared to normal liver

In order to investigate Cav-1 expression in different stages of hepatocellular carcinogenesis, we analyzed its expression profile by immunoperoxidase staining of normal liver (n = 20), cirrhosis (n = 29), and HCC (n = 95) tissue samples. The age of HCC patients ranged from 39 to 65 years (mean 53 ± 6 years). Of the 95 patients, 80 were men and 15 were women. Clinicopathological characteristics of HCC patients were summarized in Table 2.

**Table 2:** Clinicopathological characteristics of primary hepatocellular carcinoma patients.

			Caveolin-1		
				
Parameters	Variable	No. of patients	(+)	%	p*
Gender	Male	80	53	66.3	0.833
	Female	15	9	60	
Tumor size	< 5 cm	78	51	65.4	0.833
	> 5 cm	17	11	64.7	
Etiology	Hepatitis viruses	79	52	65.8	0.601
	Non-viral	11	7	63.6	
	Unknown	5	3	60	
Number of tumor nodules	< 3	43	29	67.4	0.580
	> 3	52	33	63.5	
Venous invasion	Present	38	29	76.3	0.02**
	Absent	57	33	57.9	
Tumor grade ^(a)^	Well differentiated	18	8	44.4	< 0.001**
	Moderately differentiated	60	39	65.0	
	Poorly differentiated	17	15	88.2	

* *X*^2^, ** Statistically significant

^(a) ^All of the HCC tissues used in this study are in a cirrhotic background.

**Figure 2:**
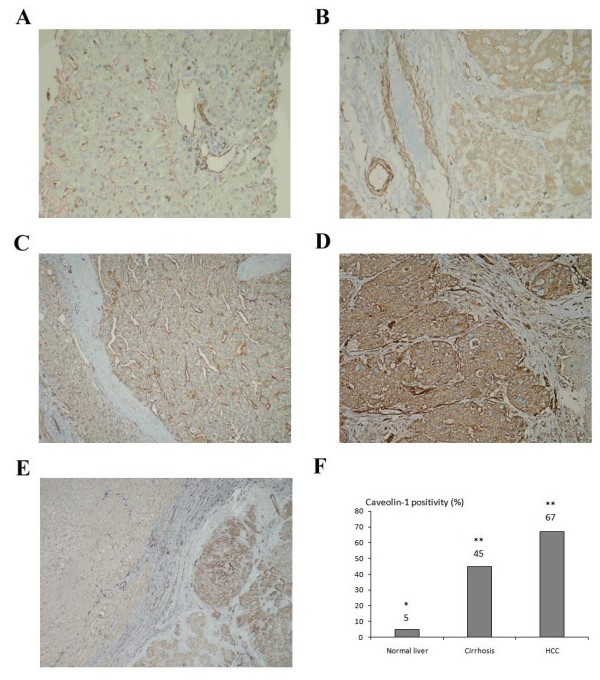
**Immunohistochemical staining of Cav-1 in normal, cirrhotic and HCC tissue sections**. **(A) **Absence of Cav-1 expression in hepatocytes of normal donor liver tissue and strong Cav-1 expression in the endothelium of the blood vessels. **(B) **Cav-1 positivity in the cirrhotic nodule and endothelium of the vessels with same intensity. **(C) **Membranous Cav-1 positivity in one of the well differentiated HCC cases. **(D) **Strong cytoplasmic Cav-1 expression in a poorly differentiated HCC. **(E) **Staining of Cav-1 in an HCC sample with its tumoral and surrounding peritumoral region. **(F) **The percentage of Cav-1 staining in normal liver, cirrhosis and HCC tissues (* p < 0.001 when HCC and cirrhotic tissues were compared to normal liver; ** p = 0.002 when HCC was compared to cirrhotic tissues). Normal liver tissues, cirrhotic tissues (without HCC) and HCC tissues are taken from different patients.

In normal liver tissues, there were very weak staining in hepatocytes for Cav-1, except one tissue that displayed moderately positive staining. In all normal tissues, endothelium of blood vessels were strongly positive, as expected (Figure 2A). In cirrhotic livers, we observed positive staining in 13 of 29 (45%) samples (Figure 2B). Finally, in 62 of 95 (65.3%) HCC samples, tumor cells stained positively for Cav-1 (Figures 2C and 2D). Out of 95 HCC samples, only 6 samples have weak caveolin staining in the tumor cells compared with its surrounding tissue. Also, caveolin-1 expression in the tumor cells was significantly stronger than the cells in surrounding tissue when analysed all HCC samples (p < 0.001) (Figure 2E). Statistical analysis of normal liver, cirrhosis and HCC data showed that the increase in cirrhosis and HCC was significant (p = 0.002 when HCC was compared to cirrhosis; and p < 0.001 when HCC or cirrhosis were compared to normal tissues) (Figure 2F).

Since we observed a close correlation between Cav-1 expression and differentiation status of HCC cell lines, we also compared Cav-1 expression with respect to differentiation status of HCC tissues. 8 of 18 (44%) well differentiated HCC tissues, 39 of 60 (65%) moderately differentiated HCCs, and 15 of 17 (88%) poorly differentiated HCCs were positive for Cav-1 (Table 2), indicating that Cav-1 expression is also closely related to differentiation status of primary HCC tumors (p = 0.001). We also noticed that, whereas majority of Cav-1 staining was membranous in well- and moderately differentiated HCC samples, the staining in poorly differentiated HCCs was strongly cytoplasmic (Figure 2C and 2D).

Besides differentiation status, Cav-1 expression was also analyzed according to other clinicopathological factors such as age, sex, portal vein invasion and etiology. A strong correlation was found between Cav-1 expression and the presence of venous invasion of HCC samples (p = 0.02), but there was no statistically significant correlation with tumor size, tumor etiology, number of tumor nodules, age and gender (Table 2). For cirrhotic tissues, Cav-1 expression showed no correlation with etiology or gender (data not shown).

### Expression of Caveolin-1 affects cell motility and invasion

**Figure 3:**
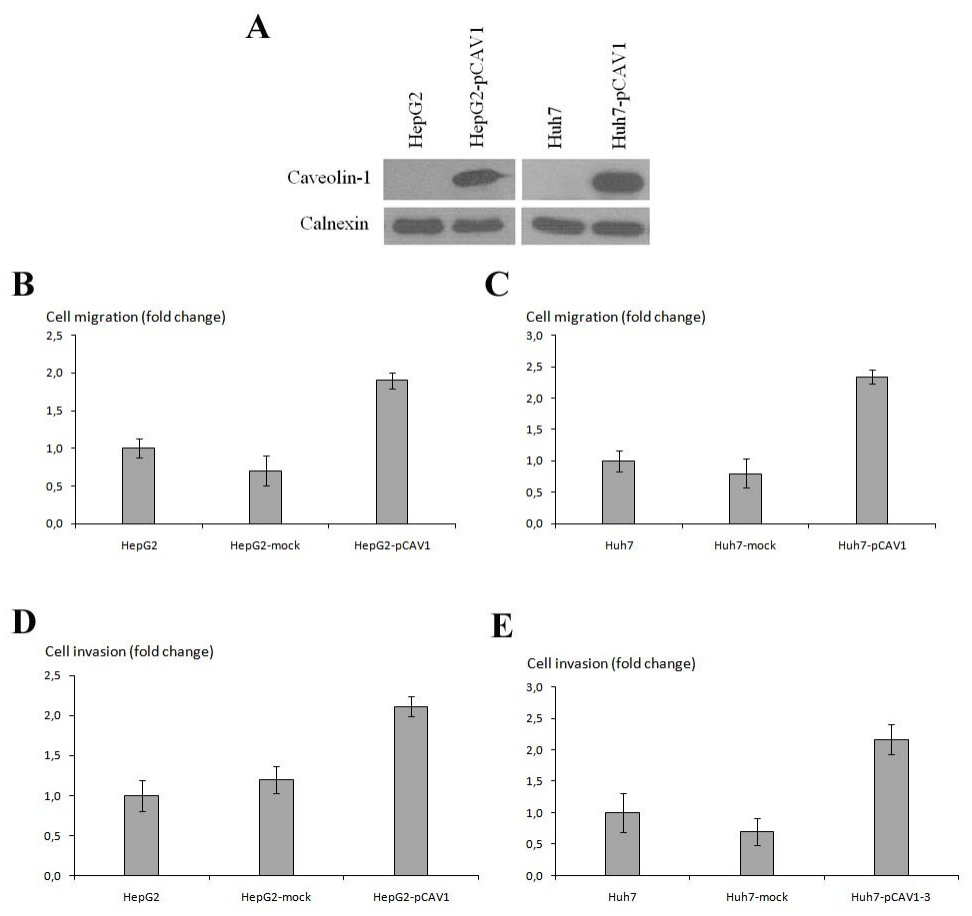
**Effect of caveolin-1 expression on the cellular migration and invasion of HCC cell lines**. **(A) **Ectopic expression of Cav-1 in Huh7 and HepG2 cells were shown by western blotting in comparison with parental cells. Stably transfected caveolin-1 producing HepG2 **(B, D) **and Huh7 **(C, E) **cells with their parental cells and vector-transfected stables clones (mock) were assayed for their ability of migration and invasion. Data includes mean ± SD values from three independent experiments and each experiment was performed as triplicate.

**Figure 4:**
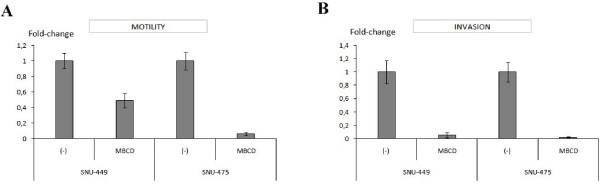
**The effect of caveolae disruption on motility and invasion of endogenously Cav-1 expressing SNU-449 and SNU-475 cells**. To determine the effect of caveolae disruption on motility and invasion of SNU-449 and SNU-475 cells, we used a cholesterol-depleting agent methyl-β-cyclodextrin (MBCD), as described in methods section. The effect of caveolae disruption on motility **(A) **and invasion **(B) **of both SNU-449 and SNU-475 cells was shown. Data includes mean ± SD values from two independent experiments and each experiment was performed as triplicate.

In order to investigate the role of Cav-1 expression on cell motility and invasion ability of HCC cells, we stably transfected pcDNA3.1/CAV1 construct into Huh7 and HepG2 cells in which endogenous Cav-1 expression was weak or negative, respectively. Cav-1-expressing clones and parental cells were analyzed in parallel by using motility/invasion chambers, as described in methods. Overexpression of Cav-1 caused approximately two fold increase both in cell motility and invasion with respect to parental and control vector transfected Huh7 and HepG2 cells (Figure 3). Moreover, motility and invasion of SNU-475 and SNU-449 cells which express Cav-1 endogenously, were analysed after caveolae structures were disrupted by addition of cholesterol depleting-agent methyl-β-cyclodextrin (MBCD) into the culture medium. Caveolae disruption caused approximately 2-fold and 10-fold decrease in the motility of SNU-449 and SNU-475, respectively (Figure 4A). Additionally, caveolae distruption caused almost complete inhibition of invasion in both SNU-449 and SNU-475 cells (Figure 4B). Thus, we concluded that Cav-1 expression in HCC cells might contribute cellular motility and invasion of these cells.

### Caveolin-1 induces both an Epithelial Mesenchymal Transition (EMT)-like phenotype and the expression of Matrix Metalloproteinases in HCC cells

**Figure 5:**
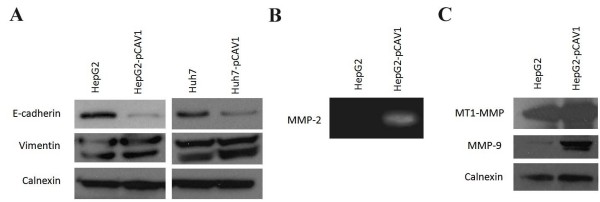
**Molecular changes in Cav-1 overexpressing Huh7 and HepG2 cells**. **(A) **Expression levels of E-cadherin and Vimentin in Cav-1 overexpressing and parental Huh7 and HepG2 cells. **(B) **Gelatin-zymography result of MMP-2 secreted to the culture supernatant of parental and Cav-1 overexpressing HepG2 cells. **(C) **Expression levels of MMP-9 and MT1-MMP in parental and Cav-1 overexpressing HepG2 cells. All western-blotting and gelatin-zymography results were confirmed with at least two independent experiments.

To determine the molecular mechanisms behind the increase in the motility and invasion under the effect of Cav-1 overexpression in HepG2 and Huh7 cells, we analysed the expression of main EMT markers in these cells. As shown in Figure 5A, E-cadherin protein level was down-regulated while Vimentin level was up-regulated in Cav-1 overexpressing Huh7 and HepG2 cells compared with parental ones. These results were consistent with the phenomena in the epithelial to mezenchymal transition (EMT). In addition, we analysed the cellular and secreted forms of Matrix Metalloproteinases (MMPs) in these cells by western-blotting and gelatin-zymography assays, respectively. Among the secreted MMPs, only MMP-2 was found as increased in Cav-1 expressing HepG2 cells (Figure 5B). Further in cell-lysates, MMP-9 and MT1-MMP were found as up-regulated in HepG2 cells overexpressing Cav-1 (Figure 5C). Taken together with EMT-phenotype, these results may explain the increased cellular motility and matrix invasion of HCC cells.

### Caveolin-1 does not affect anchorage-independent growth of HCC cells in vitro

**Figure 6:**
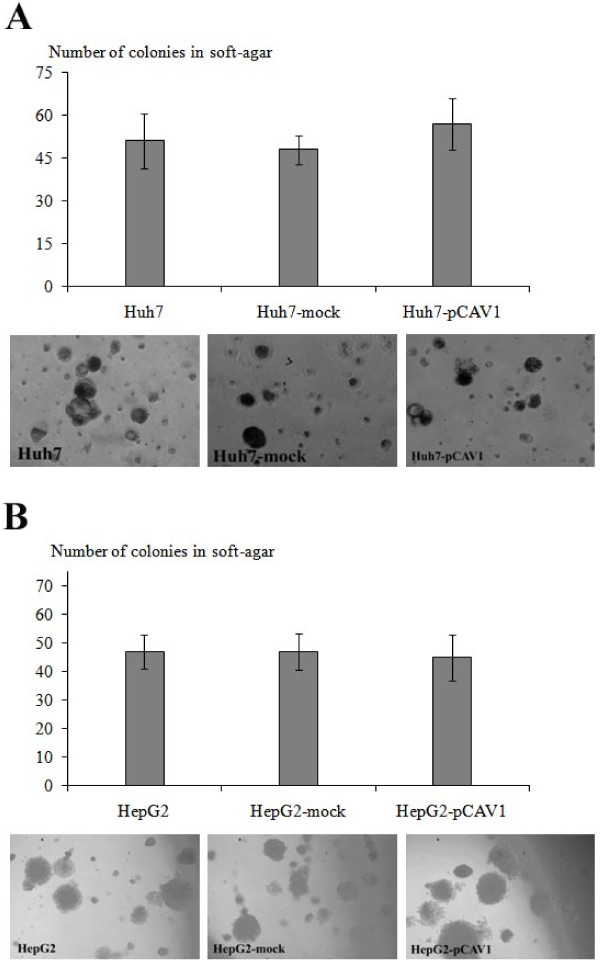
**Effect of Cav-1 overexpression on the anchorage-independent growth ability of HCC cells**. The number of colonies formed in soft agar for stably Cav-1 overexpressing, mock-transfected and parental Huh7 cells **(A) **and HepG2 cells **(B) **were shown in the graphic. Each experiment was performed in triplicate and fields are representative of three independent experiments. Colonies are photographed at 20× magnification as shown at the bottom of the graphic for each.

To evaluate the role of Cav-1 in the anchorage-independent growth in HCC cells, Cav-1-expressing clones and parental cells were used. Huh7 and HepG2 clones strongly expressing ectopic Cav-1 were analyzed for their abilities to form colonies in soft agar in comparison with their parental counterparts. Both Cav-1 expressing clones and parental cells formed high number of colonies in soft agar and there were no significant difference regarding on the colony size and the number (Figure 6).

## Discussion

Cav-1 expression was reported to be mostly stage-dependent during tumorigenesis. It is known to be absent or reduced following gene promoter-CpG-island hypermethylation in early-stage tumors, but re-expressed (by gene promoter-mCpG-DNA demethylation) in late-stages of different carcinomas such as metastasized lung, prostate, gastric carcinomas, as well as sarcomas [24]. This biphasic stage-dependent expression pattern of Cav-1 may not be valid for hepatocellular carcinogenesis. Yokomori *et al*. reported elevated expression of Cav-1 at protein and mRNA levels in a small set of cirrhotic livers (n = 5) [16]. Anders *et al*. also showed elevated Cav-1 expression in macroregenerative and dysplastic nodules which are precursor lesions of HCC [25]. Our data clearly indicate that Cav-1 is overexpressed in both premalignant cirrhotic tissues and malignant HCC tumors when compared to normal liver samples. Also, caveolin-1 is highly expressed in tumor cells more than their peritumoral cirrhotic regions in HCC tissues. This observation may be explained by different microenvironments due to the presence of tumor. Based on these data, it is strongly proven that overexpression of Cav-1 might contribute to progression of hepatocellular carcinogenesis. Thus, it can be proposed as a tumorigenesis marker for HCC.

Functional role of Cav-1 appears to differ depending on the tumor cell type and/or tumor stage. In human prostate cancers, Cav-1 expression correlates positively with extra-prostatic extension and lymph node involvement [26]. In the same study, the involvement of Cav-1 also has been clearly proven in mediating angiogenesis during prostate cancer progression. On the other hand, Williams *et al*. reported that Cav-1 is a potent suppressor of mammary tumor growth and metastasis in an animal model [27]. The functional role of Cav-1 in HCC remains elusive. In our studies, when Cav-1 was overexpressed ectopically in HCC cell lines, cells displayed increased motility and invasion. And when caveolae was disrupted by chemicals, abilities on the motility and invasion were decreased as well. Moreover, we observed a statistically significant positive correlation between venous invasion and Cav-1 expression in HCC tumor samples and showed that motility status of HCC cell lines are positively correlated with Cav-1 expressions in these cell lines. Taken together, these observations indicate that Cav-1 may functions as a promoter of motility and invasion in HCC. Additionally, we demonstrated that Cav-1 overexpression reduced proliferation of HepG2 cells whereas it did not affect proliferation of Huh7 cells (data not shown). Since Cav-1 overexpression affects cell proliferation in a cell type dependent manner, it may have a dual function in hepatocellular carcinogenesis.

The role of Cav-1 on cellular motility and invasion is not well understood in tumorigenesis. Recently, it was shown that membrane-type-1 matrix metalloproteinase (MT1-MMP) co-localizes with Cav-1 at the perinuclear region and it may be translocated to the nucleus via caveolae-mediated endocytosis in HCC [28]. A correlation between nuclear MT1-MMP and tumor aggressiveness including poor prognosis and large tumor expansion has been reported. Furthermore, Jia *et al*. demonstrated that Cav-1 expression leads to an increased production of MMP-11 and increased invasive capability in murine HCC cell lines [29]. Giannelli et al., showed that MMP2 and MT1-MMP1 expressions stimulate invasion of HCC cell lines [30]. Consistent with these studies, we showed Cav-1 overexpression caused an increase in the MMP-2 secretion to culture supernatant and in expression of MMP-9 and MT1-MMP in HCC cell-lines.

It is very well known that expressional changes from E-cadherin, an epithelial marker to Vimentin, a mesenchymal marker shows EMT phenotype for epithelial cells. EMT contributes invasive and metastatic phenotype in progression of epithelial tumors such as HCC [31]. It previously has been shown that regulation of Cav-1 plays a central role in the complex cellular changes leading to metastasis via epithelial-to-mesenchymal transition in human tumor cells overexpressing EGFR [15]. In our study, Cav-1 overexpression caused a decrease in the E-cadherin, contrarily, an increase in Vimentin expression levels. These changes are consistent with an epithelial to mesenchymal transition and may explain the motility and invasion increase observed in Cav-1 overexpressing HCC cells.

Besides contribution of Cav-1 in HCC cell motility and invasiveness, there was a strong correlation between differentiation status and Cav-1 expression in both HCC cell lines and tumor samples used in this study. We showed that poorly differentiated HCC cell-lines abundantly express Cav-1 with respect to well differentiated ones. Recently, Fuchs *et al*. classified several HCC cell-lines including which we used in this study as epithelial or mesenchymal according to their expressions of EMT markers [32]. It is proposed that caveolin-1 expression might be correlated with the status of differentiation of hepatocarcinogenesis via appearance of epithelial mesenchymal transition following high invasiveness. It is known that poorly differentiated HCC tumors are much more aggressive and metastatic when compared to well or moderately differentiated tumors [33,34]. Recently, it has also been reported that distant metastasis within 2 years after a curative hepatectomy occurred more frequently in poorly differentiated HCC group than in well or moderately differentiated HCC groups [34]. Therefore, it may be concluded that Cav-1 upregulation contributes to more invasive capability in poorly differentiated HCCs.

Another interesting result obtained in our study that Cav-1 localization in poorly-differentiated HCC tissues was strongly cytoplasmic while it was membranous in well-differentiated ones. Orlichenko *et al*. showed that caveolae internalization and cytoplasmic localization occurs only Cav-1 is phosphorylated by growth factor receptors such as EGFR [35]. So, cytoplasmic Cav-1 staining in poorly-differentiated HCC tissues shows that Cav-1 is active and takes roles in these cells. Because Cav-1 is known as a regulator for many growth factor signaling pathways and many growth factor signaling is dysregulated in HCC, Cav-1 may be a candidate regulating these signaling events.

## Conclusion

In conclusion, stepwise increase in Cav-1 expression in neoplastic stage with respect to pre-neoplastic stage during hepatocellular carcinogenesis and its ability to stimulate HCC cell motility and invasiveness indicate that this protein plays a crucial role in tumor progression. This opens new ways to use Cav-1 as a marker of tumor progression, as well as for the design of therapeutic approaches aiming to block its cellular functions. Targeting Cav-1 in HCC may represent a potential option for future HCC treatment.

## Competing interests

The authors declare that they have no competing interests.

## Authors' contributions

MC carried out western-blot, motility, invasion and anchorage-independent growth experiments; in addition, drafted the manuscript with EE. EE participated both experimental procedures and analysis of data and drafted the manuscript with MC. DN, OS and FY carried out the immunohistochemistry on tissue samples and analyzed them. MK and SK provided study material and clinical data and participated in interpretation of clinical data. NA made substantial contributions to conception and design of the study, analysis and interpretation of data, participated in writing of the manuscript and revised it critically for intellectual content. All authors read and approved the final version of the manuscript.

## Pre-publication history

The pre-publication history for this paper can be accessed here:

http://www.biomedcentral.com/1471-2407/9/65/prepub
